# Hypoxia, Inflammation and Necrosis as Determinants of Glioblastoma Cancer Stem Cells Progression

**DOI:** 10.3390/ijms21082660

**Published:** 2020-04-11

**Authors:** Marco Papale, Mariachiara Buccarelli, Cristiana Mollinari, Matteo A. Russo, Roberto Pallini, Lucia Ricci-Vitiani, Marco Tafani

**Affiliations:** 1 Department of Experimental Medicine, Sapienza University, 00161 Rome, Italy; marcopapale@hotmail.it; 2 Department of Oncology and Molecular Medicine, Istituto Superiore di Sanità, 00161 Rome Italy; mariachiara.buccarelli@gmail.com (M.B.); lriccivitiani@yahoo.it (L.R.-V.); 3 Institute of Translational Pharmacology, National Research Council, 00133 Rome, Italy; cristiana.mollinari@iss.it; 4 Department of Neuroscience, Istituto Superiore di Sanità, 00161 Rome, Italy; 5 IRCCS San Raffaele Pisana, 00163 Rome, Italy; matteoantoniorusso44@gmail.com; 6 MEBIC Consortium, San Raffaele Open University, 00166 Rome, Italy; 7 Fondazione Policlinico Universitario A. Gemelli IRCCS, 00168 Rome, Italy; roberto.pallini@Unicatt.it; 8 Institute of Neurosurgery, Catholic University School of Medicine, 00168 Rome, Italy

**Keywords:** hypoxia, glioblastoma, cancer stem cells, molecular rehabilitation, alarmins

## Abstract

Tumor hypoxic microenvironment causes hypoxia inducible factor 1 alpha (HIF-1α) activation and necrosis with alarmins release. Importantly, HIF-1α also controls the expression of alarmin receptors in tumor cells that can bind to and be activated by alarmins. Human tumor tissues possess 1–2% of cancer stem cells (CSCs) residing in hypoxic niches and responsible for the metastatic potential of tumors. Our hypothesis is that hypoxic CSCs express alarmin receptors that can bind alarmins released during necrosis, an event favoring CSCs migration. To investigate this aspect, glioblastoma stem-like cell (GSC) lines were kept under hypoxia to determine the expression of hypoxic markers as well as receptor for advanced glycation end products (RAGE). The presence of necrotic extracts increased migration, invasion and cellular adhesion. Importantly, HIF-1α inhibition by digoxin or acriflavine prevented the response of GSCs to hypoxia alone or plus necrotic extracts. In vivo, GSCs injected in one brain hemisphere of NOD/SCID mice were induced to migrate to the other one in which a necrotic extract was previously injected. In conclusion, our results show that hypoxia is important not only for GSCs maintenance but also for guiding their response to external necrosis. Inhibition of hypoxic pathway may therefore represent a target for preventing brain invasion by glioblastoma stem cells (GSCs).

## 1. Introduction

Cancer onset and progression cannot be considered a simple accumulation of mutations but a more complex process involving a wide number of molecules regulating metabolism, survival and cell proliferation [1]. In particular, the interplay between the growing cancer and its surrounding microenvironment plays a central role in influencing cancer cell behavior [2]. In this context, it is important to consider that cancer cells live in a hostile microenvironment characterized by low oxygen rate (around and below 2%) [3]. This particular condition promotes the expression of the hypoxia inducible factor 1 (HIF-1), a master key transcription factor [4]. Indeed, intratumoral hypoxia and HIF-1 are a common finding in human cancer. In particular, HIF-1 regulates processes such as angiogenesis, metabolic reprogramming, extracellular matrix remodeling, epithelial-mesenchymal transition, motility, invasion, iron homeostasis, metastasis, CSC maintenance, immune evasion, resistance to chemotherapy and radiation therapy [5]. In the case of metabolic reprogramming, hypoxia and HIF-1α induce expression of Hexokinase 2 (HK2) [6]. In both normal and cancer cells, hexokinase (HK), uses ATP to regulate the irreversible phosphorylation of glucose to glucose-6-phosphate that cannot leave the cell and is utilized as fuel for different metabolic pathways [7]. However, in cancer cells, hypoxia and other epigenetic changes, increase expression of the isoform HK2 that translocates to the mitochondria associating with components of the mitochondria permeability Transition Pore (MPTP) with the aim to efficiently produce glucose-6-phosphate for glycolysis and prevent release of cytochrome c from the mitochondria thereby inhibiting apoptosis [7]. In fact, a large number of tumors is characterized by HK2 overexpression and inhibitors of HK2 are under study as anticancer agents [8]. HIF-1 is a heterodimeric protein composed of a constitutively expressed β subunit and an O2-regulated α subunit. Under normoxic conditions, the HIF-1α subunit is synthesized and hydroxylated on proline residue 402 and/or 564 by specific prolyl hydroxylase domain (PHD) proteins (i.e., PHD2) using O2 and α-ketoglutarate as substrates to catalyze a dioxygenase reaction in which one oxygen atom is inserted into the proline residue and the other oxygen atom is inserted into α-ketoglutarate to form succinate and CO_2_. Next, the protein osteosarcoma-9 (OS-9) interacts with both PHD2 and HIF-1α, promoting hydroxylation [9]. This step is essential for the binding of the von Hippel-Lindau protein (VHL) that interacts with Elongin C and thereby recruits a ubiquitin ligase complex. This process leads HIF-1α to be degraded by the proteasome. Differently, under hypoxic conditions, the oxygen deprivation inhibits the hydroxylation reactions and/or increases mitochondrial production of Reactive Oxygen Species (ROS), which may inhibit the hydroxylases by oxidizing a ferrous ion in the catalytic site. In this way the stability and transactivation function of HIF-1α are increased, promoting dimerization with HIF-1β. Afterwards, this dimerization determines the activation of the complex and the consecutive binding to its recognition Hypoxia Response Elements (HRE) sequence 5′-(A/G)CGTG-3′ in target genes, thus increasing transcription [10,11]. Along with HIF-1 activation, hypoxic microenvironment induces necrotic cell death with release of intracellular components many of which act as damage associated molecular patterns (DAMPs) or alarmins [12]. Alarmins include ATP, proteins such as high mobility group box 1 (HMGB1), phosphatidylserine, etc. and are one of the causes of tumor-associated inflammation [13]. In fact, alarmins are “sensed” by immune, cancer, dendritic and other cells by binding to membrane receptors such as receptor for advanced glycation end products (RAGE), Toll Like Receptors (TLRs) and others [14]. In particular, RAGE is a plasma membrane receptor capable of recognizing molecular patterns on proteins such as HMGB1, S100 and β-fibrils [15,16]. Once activated, RAGE triggers a pro-inflammatory and reparative cascade involving transcription factors such as nuclear factor kappa-light-chain-enhancer of activated B cells (NF-kB) [17]. Moreover, by using tumor cell lines and Glioblastoma (GBM) tumor biopsies we have already demonstrated an HIF-dependent increase in RAGE expression [18]. Importantly, HIF-1 activation and alarmin release must not be considered as two separate and independent events. HIF-1-expressing and surviving cancer cells increase expression of alarmins receptors on their plasma membrane [19,20]. The binding of alarmins to plasma membrane receptors activates a signaling cascade that ends with the activation of the transcription factor NF-kB. At this point, cancer cells, through HIF-1 and NF-kB activation, reprogram molecular pathways of metabolism, ROS production/scavenging, autophagy, apoptosis, etc., increasing survival and resistance to stress and acquiring a more aggressive phenotype promoting tumor progression [21]. Another important and recently discovered aspect of tumors is the presence of cancer stem cells (CSCs) [22]. Importantly, CSCs reside and thrive in the hypoxic niches [23]. Indeed, hypoxia would serve as a stimulus to maintain stemness of CSCs. Moreover, CSCs represent the most resistant tumor cell population responsible for tumor relapse and metastasis [24]. Since the CSC paradigm has been developed, a number of data supporting their ability to promote tumor growth have been collected concerning different kinds of tumors such as cerebral [25], pulmonary [26], colon [27], ovary [28], pancreas [29], prostate [30] and thyroid [31], in addition to many other neoplasms, both solid and belonging to the immune-hematopoietic system [32]. However, a study detailing the effect of hypoxia, inflammation and alarmins released during necrosis on CSCs is still missing. Glioblastoma is a deadly brain tumor with a high degree of heterogeneity [33]. CSCs are present in glioblastoma in different percentages and are thought to be responsible for therapy resistance and tumor resilience [34]. Moreover, the possibility to isolate and grow, in vitro, CSCs from glioblastoma biopsies (GSCs) correlates with worse progression free survival [35]. Among GSCs that have been isolated and grown in vitro, two different clusters have emerged based on gene expression profiling, i.e., proneural-like, full stem phenotype (GSf-like) in which cells grow as neurospheres and mesenchymal-like, restricted stem phenotype (GSr-like) characterized by adherent growth in vitro [36]. In vivo, GSf-like phenotype shows a higher invasiveness than GSr-like phenotype [37].

To this direction, our hypothesis is that CSCs respond to hypoxia by increasing alarmins receptors that can bind to alarmins released by necrotic cells. By using different GSC lines, we aim to demonstrate: i) response to hypoxia by determining HIF-1α and HIF-1α-dependent gene and protein expression of vascular endothelial growth factor (VEGF) and Hexokinase (HK2); ii) gene and protein expression of alarmin receptor RAGE. Moreover, we also aim to show that, in the presence of a necrotic extract rich in alarmins, GSCs increase their invasion and adhesion capacity both in in vitro and in in vivo settings.

## 2. Results

### 2.1. HIF-1α and HIF-1α-Dependent Gene Expression in GSCs

**Figure 1:**
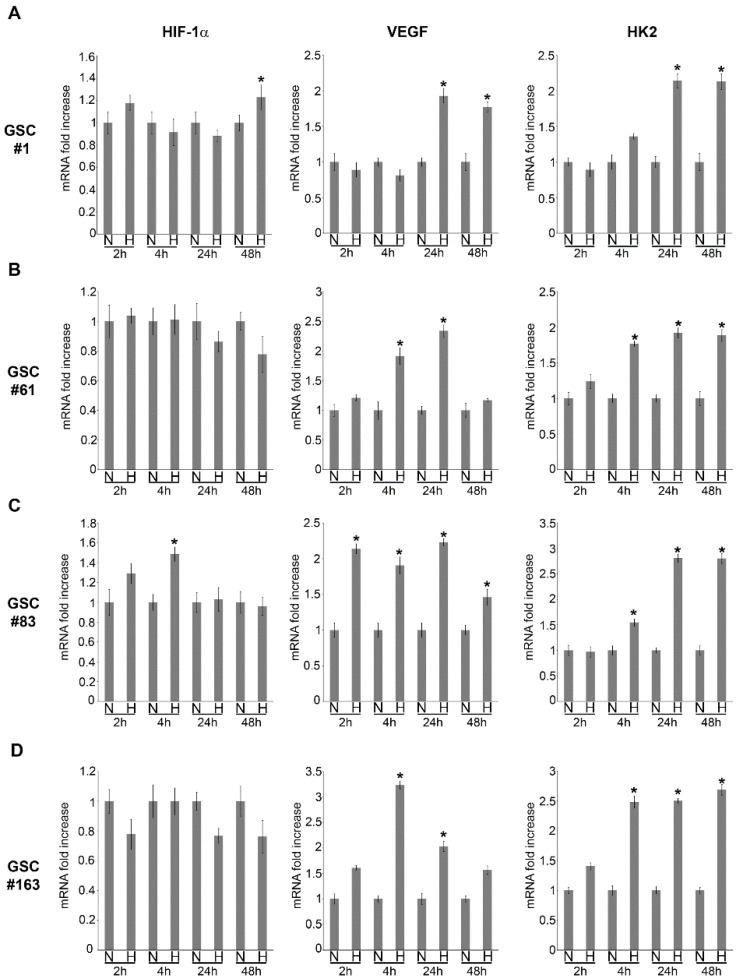
Hypoxia regulation of hypoxia inducible factor 1 alpha (*HIF-1α*), vascular endothelial growth factor (*VEGF*) and Hexokinase 2 (*HK2*) mRNA expression in glioblastoma stem-like cells (GSCs). (**A**–**D**) GSCs #1, 61, 83 and 163 were grown as indicated in Materials and Methods. Cells were then counted and plated (5 × 105) in ultra-low attachment 6-well plates and either kept in normoxic or hypoxic condition for the times indicated. Fold increase of *HIF-1α*, *VEGF* and *HK2* mRNA under hypoxia were determined by RT-PCR. Experiments in the figure were repeated three times. * Significantly different from the corresponding control normoxic cells. Significance was set at *p* < 0.05. n, normoxic cells; h, hypoxic cells.

To study how GSCs respond to hypoxia, we selected four different GSC cell lines named #1, 61, 83 and 163. These GSC lines were previously isolated and characterized [38]. In particular, we first determined *HIF-1α*, *VEGF* and *HK2* mRNA and protein expression under normoxic and hypoxic conditions. Figure 1 shows mRNA levels during a time course of 2, 4, 24 and 48 h for *HIF-1α*, *HK2* and *VEGF* under normoxia (N) and hypoxia (H) for the four GSC lines.

**Figure 2:**
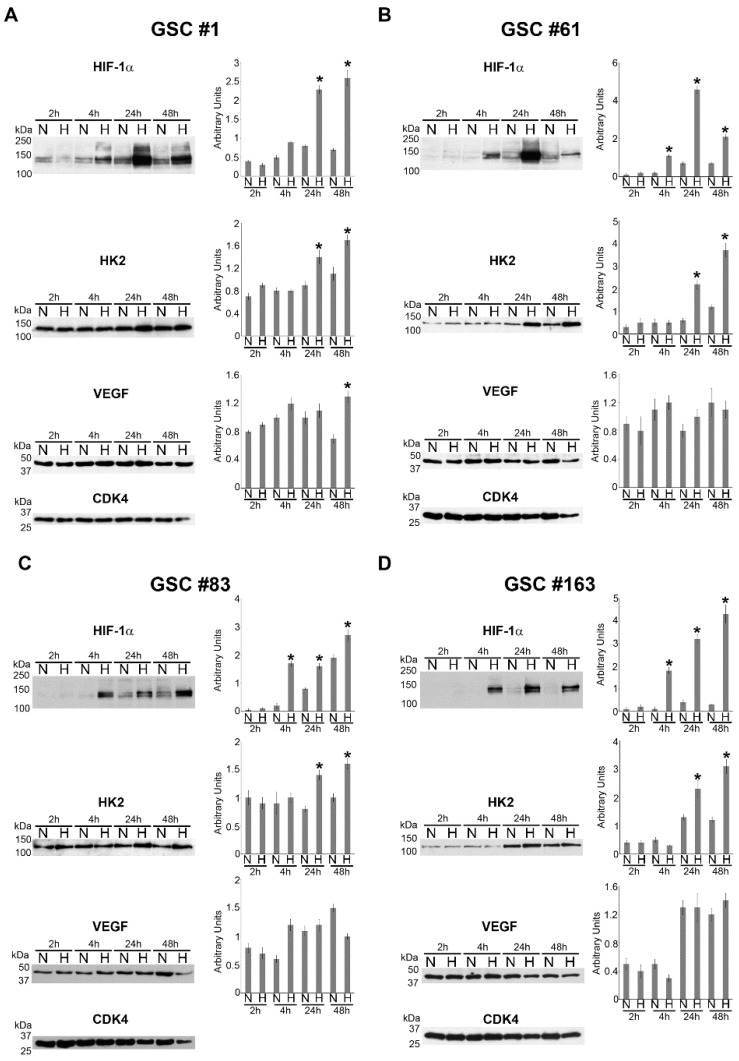
Hypoxia increases expression of HIF-1α, VEGF and HK2 in different GSC lines. (**A**–**D**) GSCs #1, 61, 83 and 163 were kept under normoxia or hypoxia for the time indicated and then processed to obtain whole cell lysates. HIF-1α, VEGF and HK2 protein expression levels were determined by Western blot as indicated in Materials and Methods. Densitometric analysis of the gels was performed by Image J software as indicated in Materials and Methods. Cyclin-dependent kinase 4 (CDK4) was used as loading control. Experiments in the figure were repeated three times. * Significantly different from the corresponding control normoxic cells. Significance was set at *p* < 0.05. n, normoxic cells; h, hypoxic cells.

In the case of GSC #1, an increase in *HIF-1α* mRNA was observed after 48 h of hypoxia whereas *VEGF* and *HK2* increased at 24 h and 48 h (Figure 1A). No mRNA increase for *HIF-1α* was observed after hypoxia for GSC #61 whereas, *VEGF* and *HK2* mRNA increased after 4 and 24 and 4, 24 and 48 h, respectively. (Figure 1B). In GSC #83 we observed an increase in mRNA expression for *HIF-1α* after 4 h of hypoxia. *VEGF* mRNA showed an increase during hypoxia treatment whereas *HK2* mRNA increased after 4, 24 and 48 h of hypoxia (Figure 1C). Finally, in GSC #163, we, again, did not observe an increase of *HIF-1α* mRNA. By contrast, we observed a mRNA increase for *VEGF* following 4 and 24 h hypoxia and for *HK2* after 4, 24 and 48 h of hypoxia (Figure 1D). These results were confirmed by measuring protein expression by Western blot. As shown in Figure 2A, GSC #1 responded to hypoxia by increasing HIF-1α protein, an effect that started after 4 h and continued up to 48 h.

The increase of HK2 and VEGF was instead significant after 24 and 48 h of hypoxia (Figure 2A). GSC #61 showed an increase of HIF-1α protein after 4 h of hypoxia that continued at 24 and 48 h (Figure 2B). This was accompanied by an increase of HK2 after 24 and 48 h of hypoxia. However, no changes for VEGF were measured (Figure 2B). GSC #83 showed an increase in HIF-1α expression after 4, 24 and 48 h of hypoxia. HK2 expression increased after 24 and 48 h of hypoxia (Figure 2C). No change for VEGF was observed. Finally, also for GSC #163 we observed an increase of HIF-1α from 4 to 48 h and an increase of HK2 that started at 24 h and was maintained after 48 h of hypoxia (Figure 2D). Again, VEGF did not show any change in expression. Altogether, these data show that the four GSCs respond to hypoxia by stabilizing HIF-1α protein during the first 4 h. However, such a response is sustained by de novo HIF-1α protein synthesis when hypoxia is prolonged up to 24 h. On the other hand, HIF-dependent genes herewith investigated are upregulated starting at 24 h hypoxia exposure and show differences in expression among the four GSCs.

### 2.2. Hypoxia-Dependent Expression of the Alarmin Receptor RAGE

**Figure 3:**
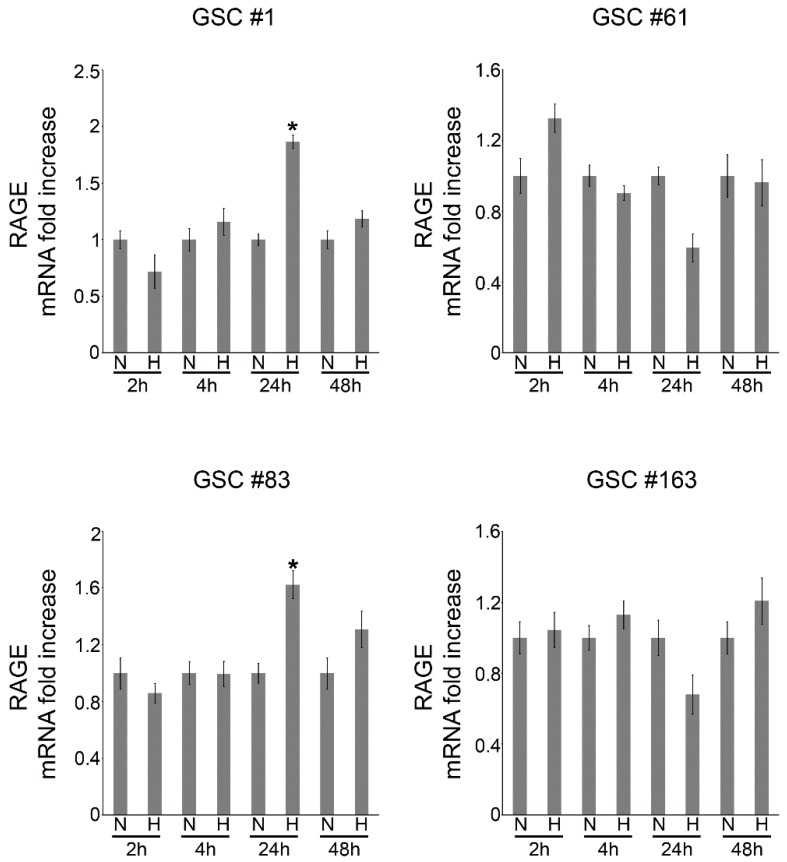
Hypoxia regulation of receptor for advanced glycation end products (RAGE) mRNA expression in GSCs. GSCs #1, 61, 83 and 163 were grown as indicated in Materials and Methods. Cells were then counted and plated (5 × 10^5^) in ultra-low attachment 6-well plates and either kept in normoxic or hypoxic condition for the times indicated. Afterward, fold increase of RAGE mRNA under hypoxia was determined by RT-PCR. Experiments in the figure were repeated three times. * Significantly different from the corresponding control normoxic cells. Significance was set at *p* < 0.05. n, normoxic cells; h, hypoxic cells.

Our next step was to measure the expression of RAGE in the four GSCs under normoxic and hypoxic conditions. As stated before, RAGE was chosen because of our previous studies confirming an HIF-dependent increase in RAGE expression [18]. As shown in Figure 3, compared to the normoxic control, RAGE expression increased after 24 of hypoxia in two of the four GSCs examined, namely in GSCs #1 and #83.

**Figure 4:**
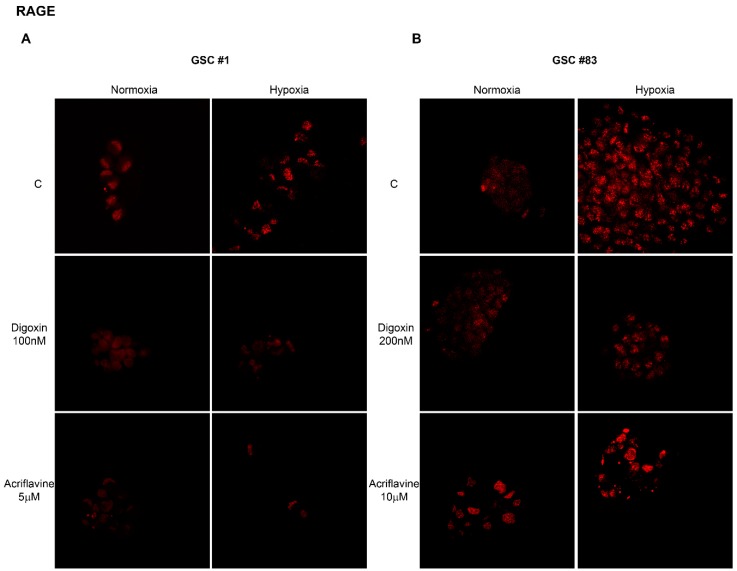
Hypoxia-induced RAGE expression is prevented by HIF-1α inhibition. (**A**) GSC #1 was grown on glass coverslips as indicated in Materials and Methods. Afterward, cells were kept under normoxia or hypoxia for 24 h in the presence or absence of 100 nM Digoxin and 5 µM Acriflavine and then processed for RAGE immunofluorescence staining as indicated in Materials and Methods. Images were taken with a Zeiss LSM 510 confocal microscope (Magnification 60×). (**B**) GSC #83 was grown on glass coverslips as indicated in Materials and Methods. Afterward, cells were kept under normoxia or hypoxia for 24 h in the presence or absence of 200 nM Digoxin and 10 µM Acriflavine and then processed for RAGE immunofluorescence staining as indicated in Materials and Methods. Images were taken with a Zeiss LSM 510 confocal microscope (Magnification 60×).

Therefore, considering the results of Figure 1; Figure 2 we decided to continue the studies by using GSCs #1 and 83. To study HIF-1α-dependent RAGE expression, GSCs #1 and 83 were kept under normoxic or hypoxic conditions in the presence or absence HIF-1α inhibitors. In fact, several natural and synthetic compounds have been discovered and used to inhibit HIF [39]. Among those, herewith, we choose Digoxin and Acriflavine that have been shown to prevent HIF-1α protein synthesis and dimerization, respectively [40,41]. Results of these experiments are reported in Figure 4 showing an increase of RAGE expression under hypoxia that was prevented by treating GSC #1 with 100 nM Digoxin and 5 µM Acriflavine and GSC #83 with 200 nM Digoxin and 10 µM Acriflavine. The results shown in Figure 4 have been summarized in Table 1 where we reported the mean fluorescent intensity per cell. It must be noted that the reduction of RAGE fluorescent observed after treating hypoxic GSCs with either Digoxin or Acriflavine in most probably due to the combined effect of both a reduction of RAGE expression and of the number of cells. In fact, as also observed by us (not shown) and previously reported, both Digoxin and Acriflavine reduce tumor cell growth both in vitro and in vivo [40,41].

The different doses were determined following previous published results on other cancer cell lines [40,41] and by treating GSCs #1 and #83 with Digoxin from 50 to 200 nM and with Acriflavine from 2.5 to 10 µM and measuring HIF-1α expression as shown in Supplementary Figure S1. Moreover, Figure 4 shows: (i) the different growth behavior of GSC #83 compared to GSC #1 that also justify the different doses of Digoxin and Acriflavine used in order to reach the center of the neurosphere; (ii) the uneven and patched cellular distribution of RAGE in the cells after hypoxia treatment.

### 2.3. GSC Migration and iInvasion in the Presence of Necrotic Extracts

**Figure 5:**
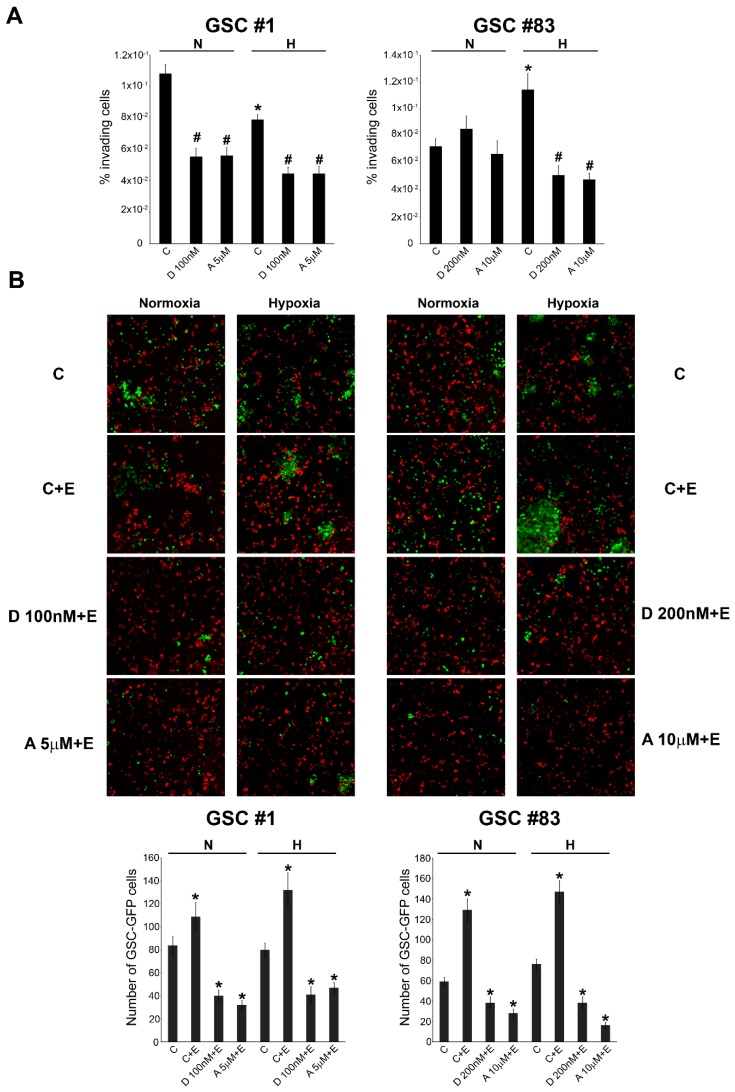
Invasion and adhesion of GSCs is influenced by hypoxia. (**A**) GSCs #1 and 83 were plated in the inserts of an invasion chamber plate as described in Materials and Methods. The invasion chamber was then incubated for 24 h in normoxic (N) or hypoxic (H) condition in the presence or absence of digoxin or acriflavine. Moreover, a necrotic extract (1 µg/mL) was placed at the bottom of each well. Invading cells were stained as described in Materials and Methods and then visualized with an optical microscope and counted. Graph represents the percentage of invading cells from a total of 150.000. C, control untreated cells; D, Digoxin, A, Acriflavine. Experiments in the figure were repeated three times. * Significantly different from the corresponding control normoxic cells. # Significantly different from the corresponding control normoxic or hypoxic cells. Significance was set at *p* < 0.05. (**B**) Human umbilical vein endothelial cells (HUVEC) were stained with Calcein Red AM and seeded on a glass coverslip in the presence or absence of a necrotic extract for 24 h as described in Materials and Methods. Afterward, GFP-GSCs #1 and 83, previously kept under hypoxia in the presence or absence of Digoxin and Acriflavine, were plated on such a layer. After 24 h, non adherent GFP-GSCs #1 and 83 were removed by extensive washing. GFP-GSC were then visualized by confocal microscopy (Magnification 60×). Images were then taken and analyzed by Image J software. Experiments in the figure were repeated three times. * Significantly different from the corresponding normoxic or hypoxic control cells. Significance was set at *p* < 0.05. C, control untreated cells; D, Digoxin, A, Acriflavine; E, Necrotic Extract.

The increased RAGE expression observed under hypoxia, could serve in GSCs as a receptor for “sensing” the presence of a necrotic stimuli coming from cells dying because of reduced oxygen tensions. To this effect, we performed migration and invasion assays. In these experiments, GSCs were kept under normoxic and hypoxic conditions in the presence or absence of HIF-1α inhibitors digoxin or acriflavine as previously described. Afterward, cells were plated in invasion chambers as described under Methods. Necrotic extracts were placed in the lower side of the chamber as a chemo-attractant to stimulate cellular invasion. Results are reported in Figure 5A and show a different behavior between GSCs #1 and #83.

**Figure 6:**
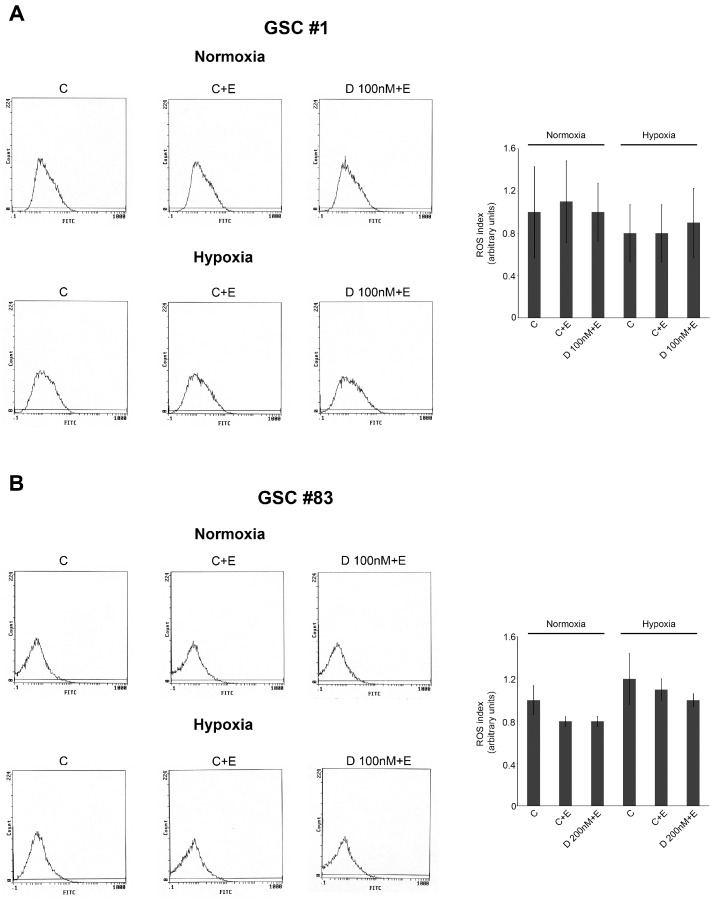
ROS production in normoxic and hypoxic GSCs. (**A**,**B**) GSCs #1 and 83 were seeded in a 6-well plate. The day after, cells were either left untreated or treated necrotic extracts with or without 100 nM digoxin as indicated in the Materials and Methods. Afterward, plates were kept under normoxia or hypoxia for 24 h. ROS production was assessed by 2′,7′-dichlorfluorescein-diacetate (DCFH-DA) staining and fluorescence intensity measured by flow cytometry.

In fact, in GSC #1 we observed a decrease of the percentage of invading cells under hypoxia compared to normoxia. Importantly, HIF-1α inhibition by digoxin or acriflavine inhibited GSC invasion both in normoxic and hypoxic conditions. By contrast, in GSC #83 hypoxia increases migration and invasion compared to normoxia. Again, Digoxin and Acriflavine inhibited migration and invasion of GSC #83 but only in hypoxic conditions (Figure 5A). Next, we also assessed the ability of GSCs to adhere to HUVEC cells in a co-culture experiment. For this experiment, we used GFP-GSCs whereas HUVEC were stained with Red Calcein-AM. In order to individuate if necrotic cell death could increase the number of cancer stem cells adhering on a HUVEC substrate, the experiment was conducted in the presence or absence of necrotic extracts. Moreover, GFP-GSCs were kept under normoxia or hypoxia for 24 h before proceeding to the co-culture experiment. As shown in Figure 5B, in normoxia, the presence of a necrotic extract increased the number of GFP-GSC cells attached to the HUVEC monolayer. Interestingly, the addition of both Digoxin and Acriflavine decreased such a number. However, when GSCs were kept under hypoxia, the number of adhering cells was higher than normoxia in particular in the presence of necrotic extracts (Figure 5B). In addition, in this case, a reduction of adhering GSCs was obtained after treating GSCs #1 and #83 with both Digoxin and Acriflavine. We also determine if the presence of necrotic extracts and or hypoxia could influence ROS production in GSCs cells. Figure 6 shows that there were no significant changes in ROS content in both GSCs #1 and #83 when necrotic extract was added both under normoxia or hypoxia.

Finally, our results on Figure 5 suggest that: (i) when in the presence of a necrotic stimulus, GSCs increase their invasion and adhesion abilities; (ii) hypoxia increases such a response.

### 2.4. In Vivo GSC Migration

**Figure 7:**
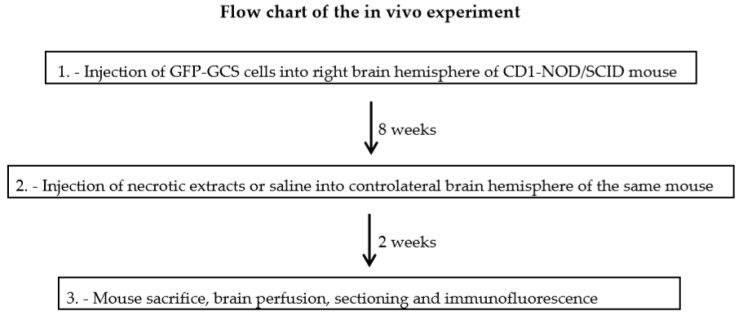
Flow chart of the different steps of the in vivo experiments.

We then examined migration of GSCs through the brain of SCID mice in the presence of necrotic extracts. In particular, we aimed to test the ability of GSCs to be attracted and to move towards an inflamed substrate. The steps of the in vivo experiments are schematized in the flow chart of Figure 7.

**Figure 8:**
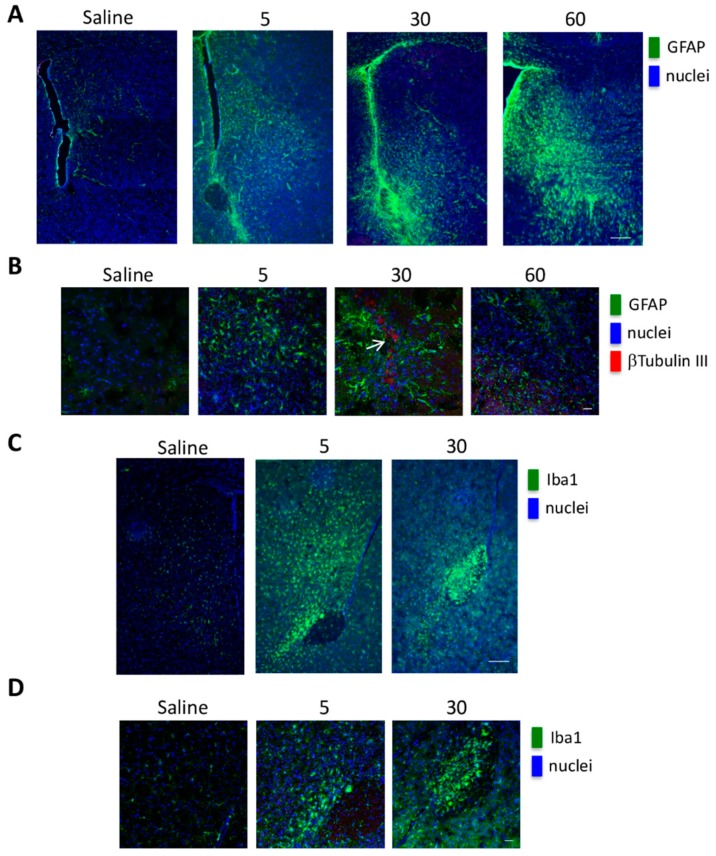
Astrocytes and microglia activation following in vivo administration of necrotic extracts. Images of CD1 Nonobese diabetic/severe combined immunodeficiency (NOD/SCID) mouse brains inoculated in one hemisphere with saline or different concentration of necrotic extracts. (**A**) Double immunofluorescence images of brain slices labeled with an anti-Glial fibrillary acidic protein (GFAP) antibody (green) and nuclei counterstained by Hoechst (blue), show an increase in GFAP labeling, due to the presence of activated astrocytes, only in the hemispheres receiving the necrotic extract, as compared with the hemispheres receiving only saline. Scale bar 200 μm. (**B**) Triple immunofluorescence images of mouse brain sections stained with anti-GFAP (green) and anti-βTubulin III (red) primary antibodies and Hoechst as nuclear counterstain. Following the necrotic treatment, it is possible to observe a recruitment of activated astrocytes (GFAP + cells) and in some cases of βTubulin III + cells (white arrow), probably representing resident neural progenitor cells attracted by the necrotic stimulus. Scale bar 20 μm. (**C**,**D**) Double immunofluorescence images of mouse brain sections stained with anti-Iba1 antibody (green) and Hoechst as nuclear counterstain (blue), show the presence of activated microglia only in the hemisphere receiving necrotic extracts. Scale bar 20 μm.

Initially, we tested the pro-inflammatory effects of 5, 30, 60, 100 ng/5 µL cellular necrotic extracts. A dose dependent activation of astrocytes, as assessed by Glial fibrillary acidic protein (GFAP) immunostaining, was detected in the brain region that received the necrotic extracts (Figure 8A,B).

**Figure 9:**
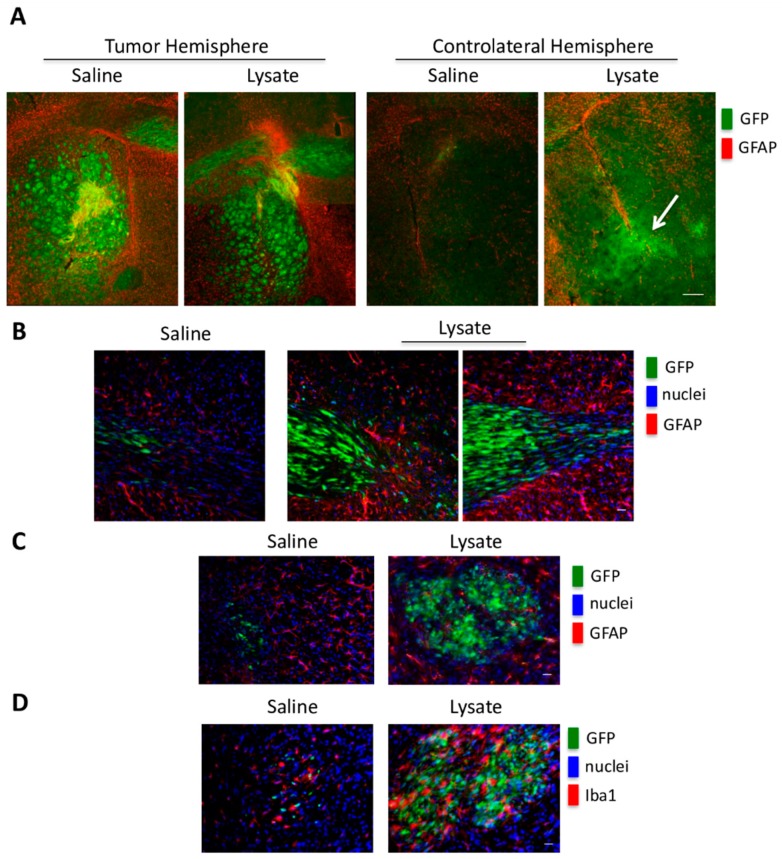
Brain migration of GFP-GSCs elicited by necrotic extract. GFP-GSC #1 cells were injected in the right brain hemisphere of CD1 NOD/SCID mice. After 8 weeks, necrotic extracts (30 ng/5 μL) were injected in the controlateral brain hemisphere. Brain sections were taken two weeks later as described in Materials and Methods. (**A**) Triple immunofluorescence images of mouse brain slices showing inoculated GFP + cells, GFAP + cells and nuclei counterstained with Hoechst. In the left panel, it is evident the growth of a GFP-GSC #1 tumor mass (green) surrounded by GFAP positive astrocytes (red). Right panel, the hemisphere, controlateral to the tumor mass, shows an increase in GFAP labeling only in the presence of necrotic extract (white arrow), as compared to the controlateral hemisphere receiving saline treatment. Scale bar 200 μm. (**B**) Triple immunofluorescence images show an increase of migration, through the anterior commissure, of inoculated GFP-GSC cells from the side of tumor formation towards to side receiving the necrotic stimulus. Interestingly, the GFP-GSC cells have an elongated morphology suggesting a migratory behavior and appear surrounded and limited by GFAP positive cells. Scale bar 20 μm. (**C**,**D**) Triple immunofluorescence images of the controlateral hemisphere show an increase in the number of GFP-GSCs and the presence of Iba1 positive microglia cells mixed within the migrated tumoral cells. Scale bar 20 μm.

In the same brain region, we observed an increase of ionized calcium binding adaptor molecule-1 (IBA1), suggesting microglia activation in the presence of necrotic extracts (Figure 8C,D). Based on these results, we choose the concentration of 30 ng/5 µL of necrotic extracts for the brain xenografts. In these experiments, GFP-GSC #1 cells were injected in the right brain hemisphere. After 8 weeks, necrotic extracts were injected into the controlateral hemisphere. In control mice, saline solution was injected in the left brain hemisphere. Collection and analysis of the brains was performed two weeks later. Extensive tumor growth was observed in the right hemisphere of each inoculated brain (Figure 9A, left panel), and in the brain hemisphere of the mice treated with the necrotic extracts, we found an extensive invasion by GFP-GSCs (Figure 9A, left panel).

Interestingly, brain migration was particularly remarkable at the level of the anterior commissure (about a 5 fold increase in GFP labeling intensity), where GFP-GSCs showed an elongated phenotype, typical of migrating cells and appeared surrounded by GFAP positive cells (Figure 9A,B). Moreover, into the controlateral hemisphere, we observed the presence of IBA1 positive cells, mainly distributed inside the migrated GFP-GSCs mass, as compared to GFAP positive cells (Figure 9C,D). Our results indicate that the presence of inflammation or necrosis represents a potent stimulus favoring GSC migration and dissemination inside the brain.

## 3. Discussion

Hypoxia and necrosis, with subsequent microenvironment inflammation, can be considered two main features of growing tumors and for this reason, the hypothesis driving this study is that these two events play a major role in determining the metastatic potential of CSCs present in a tumor. Thus, in cancer stem cells from a primary tumor, hypoxia may link the expression of genes of the inflammatory reparative response (IRR) to tumor progression. Therefore, progression of transformed CSCs does not necessarily require new genetic mutations but can be obtained through a sequential activation of hypoxia adaptation and proinflammatory genes expression. Several studies recognized hypoxic microenvironment and HIF-1 activation as fundamental regulators of cellular adjustment in this particular context [5,42]. Moreover, we and others have previously shown that GBM tumor biopsies are largely hypoxic, present necrotic areas and have a high HIF-1 and HIF-target genes expression [18,43,44,45]. Herewith we studied the response of GSCs to two components of tumor microenvironment, i.e., hypoxia and necrosis, the latter strictly associated with tissue inflammation. First of all, our results have shown substantial differences between GSC lines not only in protein expression but also in mRNA concerning HIF-1 and other important markers such as VEGF or HK2, confirming the heterogeneity of this kind of tumor [46]. Thus, molecular adaptation to hypoxia has been assessed in four different human GSCs by RT-PCR and Western blot protein analysis. A significant increase of HIF-1α and other HIF-1 target genes has been gradually observed. Indeed, in tumor cells, a great number of adaptations occurs when they are subjected to hypoxia in tumor microenvironment, and these changes regard metabolism, oxygen distribution, etc. [47,48]. In fact, we documented modifications of HK2 or VEGF expression especially in line #1 and in line #83 whereas in line #61 and in line #163, these modifications were less clear. A peculiar behavior was observed in VEGF in line #83. In fact, in this GSC line, we observed a modulation of VEGF mRNA by hypoxia that was not paralleled by a modulation of VEGF protein expression (compare Figure 1C with Figure 2C). Even if we did not investigate into that, we think that such a discrepancy might be due to: (i) a post-transcriptional control by miRNA that prevent expression of VEGF from mRNA either directly as in the case of miR-16 [49] or indirectly by targeting VHL protein as in the case of miR-566 [50]; (ii) the intratumoral heterogeneity of line #83 that contains tumor cells with different metabolic behavior that when sub-cloned gave rise to cell lines belonging to separate metabolic clusters [51]. In the latter case, this heterogeneity might reflect in opposite trends of VEGF protein expression showing, on average, no significant increase.

Overall, our results are in accordance with scientific data confirming the main activity of HIF-1 as master regulator in the complicated process of cellular adaptation to tumor microenvironment [42,48]. However, our interest was in determining the expression of plasma membrane receptor that would allow GSCs to sense the presence of alarmins or DAMPS released by necrotic cells. To this effect our results showed that RAGE mRNA and protein levels increased in GSCs following hypoxia exposure. Indeed, one of the main features of tumor cells is that they live in a hostile microenvironment characterized also by high levels of inflammation caused by necrosis and subsequent release of alarmins [19]. In order to survive and thrive in such a microenvironment tumor cells modify, among many other molecules and pathways, their ability to sense external stimuli and eventually, to migrate towards new sites [52,53]. The expression of RAGE as well as other receptors in the presence of a hypoxic microenvironment has been observed in many tumors and represents an important step for tumor progression [54,55]. Indeed, immunofluorescence analysis revealed an increased expression and different distribution of RAGE as result of hypoxia treatments (Figure 3 and Figure 4). Interestingly, treatment of GSCs with HIF-1 inhibitors such as digoxin and acriflavine, determined a decrease of RAGE expression suggesting a hypoxia- and HIF-1α-dependency of RAGE in GSCs. In fact, HIF-1α-dependent RAGE expression has been documented in many pathological situations [55,56,57,58]. In addition, hypoxia increases GSCs migration and invasion toward an attractive stimulus represented by a necrotic extract as well as their ability to adhere to HUVEC cells (Figure 5). Invasion assays revealed that, at least for GSC #83, number of invading cells was increased by hypoxia and prevented by digoxin and acriflavine treatment (Figure 5). On the other hand, our results indicate that GSC-GFP adhesion to a HUVEC layer is stimulated by hypoxia and further increased if HUVEC are previously treated with a necrotic extract. In this case, a remarkable difference can be observed between normoxic and hypoxic conditions with the latter increasing the number of adhering GSCs. Moreover, the addition of necrotic extract to HUVEC determined an increase of GFP-GSCs (Figure 5). Importantly, digoxin and acriflavine reduced the number of adhering GFP-GSCs on the HUVEC surface, both in normoxia and in hypoxia conditions, suggesting a connection between hypoxia, HIF-1α expression and extracellular signals coming from necrosis as well as necrosis-associated modification of HUVEC. Indeed, modification of endothelial cells in blood vessels is induced by inflammation associated to necrosis and may represent an important stimulus for cancer cell adhesion during tumor progression and metastasis formation [59]. In fact, necrosis, inflammation and HIF-1 activation not only promote modifications in cell metabolism or angiogenesis, but also induce tumor cell invasion [5,60,61]. Hypoxic tumor microenvironment may be linked to a considerable production of ROS which influences cellular fate. Indeed, high levels of ROS could determine an interference in the HIF-1 regulation process, promoting the dimerization of HIF-1α with HIF-1β, leading to a consecutive process activation and binding to HRE in target genes [62,63]. The increased HIF-1 activation, in turn, determines a constant and progressive alteration of the environment that leads to a complete destabilization. Thus, we measured ROS levels in GSCs #1 and #83. An important aspect was that the addition of necrotic extracts did not increase ROS levels in both GSCs #1 and #83 suggesting that this is not the main mechanism. It is worth noting that there is a strong heterogeneity among GSCs derived from different patients that accounts for differences in tumor behavior and therapy response [64]. In fact, as previously documented, GSCs #1 and 83 show different metabolic profiles [51]. Finally, we also performed in vivo analysis to validate our in vitro results. First, we determined the optimal concentration of necrotic extract sufficient to induce an inflammation without affecting mouse health. Such a concentration was set at 30 ng/5 µL (Figure 7). Inflammation was determined by observing increased activation of astrocytes and microglia assessed, in the mouse brain, through GFAP and IBA1 staining, respectively (Figure 7). Indeed, both GFAP and IBA1 are considered and used to demonstrate activation of astrocytes and microglia, respectively, during inflammatory events or diseases such as tubercolus meningitis, astrogliosis, etc. [65,66]. Next, we proceeded to evaluate if the inflammation stimulus induced by necrotic extracts could influence GSC migration compared to the saline solution alone. Indeed, after tumor growth, we observed an increase in GFAP expression only in the brain of mice receiving necrotic extracts (Figure 8). Furthermore, we also demonstrated cellular specific morphologic modifications tracing GFP-GSCs moving toward the inflamed controlateral hemisphere probably through the anterior commisure. Finally, a different distribution of astrocyte and microglia has been revealed. Indeed, GFAP positive cells distributed around GFP-GSCs whereas IBA1 positive cells were found among GFP-GSCs, probably due to the different role and interaction with tumor cells of astrocytes and microglia. It is known that inflammation promotes tumor cell proliferation and increases GBM invasion [67], moreover, inflammation after surgical resection of GBM can increase tumor progression of remaining tumor cells [68,69].

### Limitations and Considerations

We realized that our study was limited by the intrinsic heterogeneity of GSC lines. In fact, in terms of RAGE expression we noticed that only 2 of them had a significant increase following hypoxia exposure. In addition, we unraveled differences also between these two selected GSCs. We think that the reasons were: (i) the response to hypoxia was different between these two cell lines in term of HIF-1α protein expression showing, cell line #1, stronger expression than cell line #83; (ii) these two GSC lines are representative of the two GSC clusters, in particular GSC #1 shows the proneural-like phenotype (GSf-like) growing as neurospheres in vitro, whereas GSC #83 shows the mesenchymal-like phenotype (GSr-like) characterized by adherent growth in vitro [36]. It is known that these two clusters have differential expression of a large number of genes and metabolic profiles [51]. Moreover, the results on the four GSC first and on the selected two later, indicate the importance of the patient’s tumor history from transformation to progression and the need for a personalized characterization that can be achieved not only genetically or metabolically but also by measuring alarmin receptor expression and response to inflammation in the CSCs as suggested in this work. In fact, a connection between the hypoxia present in the growing GBM and the inflammation-related stimuli coming from both the tumor-associated necrosis or originating from other brain regions, is still missing. To this effect, in vitro and in vivo results, herewith reported, indicate that such connection requires the expression of HIF-1α-dependent membrane receptors with an increase of tumor cancer cell migration and invasion.

## 4. Materials and Methods

### 4.1. Cell Lines and Hypoxia Treatments

Glioblastoma stem-like cells (GSCs) were isolated from surgical samples of adult GBM patients (WHO grade IV) who had undergone complete or partial surgical resection at the Institute of Neurosurgery, Catholic University School of Medicine in Rome, upon patient informed consent and approval by the Institutional Ethics Committee of Fondazione Policlinico Gemelli, UCSC (Prot. 4720/17) as previously described [35,38]. GSC cultures were established from surgical specimens through mechanical dissociation and culturing in serum-free medium supplemented with 20 ng/mL epidermal growth factor (EGF) and 10 ng/mL basic fibroblast growth factor (βFGF) (Peprotech, Rocky Hill, NY, USA). Under these conditions, GSCs actively proliferate requiring 3–4 weeks to establish cell lines. GSCs grow as spheroid clusters (tumorspheres) expressing stem cell markers, such as CD133, sex determining region Y-box 2 (Sox2), Musashi-1 and Nestin [35,38]. The in vivo tumorigenic potential of GSCs was assessed by intracranial or subcutaneous cell injection in immunocompromised mice, resulting in tumors with the same antigen expression and histological tissue organization as the human parent tumor. Four GSC lines (GSCs #1, #61, #83, #163) obtained from surgical specimens were used in this study. Hypoxia was performed using a Hypoxic chamber (Modular Incubator Chamber, Billups-Rothenberg, Del Mar, CA, USA). Cells were seeded in appropriate plates (6-well plate with lid, flat bottom, ultra-low attachment surface, Costar) in the hood, then inserted, without lid, inside the chamber in which a 1% oxygen gas solution has been fluxed in for 4 min. The sealed chamber was placed in an incubator at 37 °C in 5% CO_2_ during the treatment. Where indicated, the same cells have also been treated with digoxin (Sigma-Aldrich, Milan, Italy) and acriflavine (Sigma-Aldrich).

### 4.2. Necrotic Cell Extracts

Untreated GSCs have been used to obtain necrotic extracts. It is worth stressing, that, for us, it is not important to know the exact composition of the necrotic extract. In fact, such necrotic extract can vary from one cell type to another still maintaining its biochemical properties that are due to the presence of alarmins. What is important is to demonstrate a general principle: necrosis is always accompanied by release of alarmins that are recognized by surrounding cells through a series of different membrane receptors that trigger an intracellular molecular signaling modifying cellular behavior. Briefly, cells have been washed once or twice in phosphate buffered saline (PBS) (Sigma Aldrich) and the pellets collected. For the preparation of whole cell extracts, cells were resuspended in a hypotonic solution 10 mM Tris–Cl pH 8.0, 1 mM KCl, 1.5 mM MgCl2, 0.1 mM DTT. Cells were then broken through a “freeze and thaw” protocol (1 min in liquid nitrogen followed by 1 min at 37 °C, 4 times). Protein content was determined by Bradford assay using BSA as standard (Biorad) and samples were stored at −80 °C.

### 4.3. SDS-PAGE and Western Blot Analysis

Cells were centrifuged at 800× *g* for 10 min at 4 °C and pellet were resuspended in 100 μL of a solution containing 50 mM Tris-Cl (Sigma-Aldrich), 250 mM sodium chloride (NaCl; Sigma-Aldrich), 5 mM ethylene diamine tetraacetic acid (EDTA; Sigma-Aldrich), 0.1% Triton^®^ X-100 and 0.1 mM Dithiothreitol (DTT, Sigma-Aldrich) plus 1 mM phenylmethylsulfonyl fluoride (PMSF; Sigma-Aldrich), Protease inhibitor cocktail (PI; Sigma-Aldrich), 1 mM sodium orthovanadate (NA3VO4; Sigma-Aldrich) and 10 mM sodium fluoride (NaF; Sigma-Aldrich) (lysis buffer). After 10 min on ice, samples were centrifuged at 14,000× *g* for 10 min at 4 °C and the supernatants were collected. Protein concentration was determined by the Bradford assay (Bio-Rad, Milan, Italy). Clarified protein lysates (40 μg) were boiled for 5 min, electrophoresed onto denaturating SDS-PAGE gel and transferred onto a nitrocellulose membrane (Bio-Rad). The blotting membranes were blocked with 5% non-fat dry milk (Bio-Rad) for 1 h RT and then incubated with primary antibody overnight at 4 °C. Antibodies used were as follows: HIF-1α (BD Transduction Lab), VEGF (Abcam, Cambridge, UK), Hexokinase 2 (Santa Cruz Biotechnology, Heidelberg, DE), CDK4 (Santa Cruz Biotechnology). The following day, membranes were washed three-times with 0.1% Tween^®^ 20 (Sigma-Aldrich) in PBS (PBST) for 30 min at RT and incubated with the appropriate secondary antibody for 1 h at RT. After another 3 washes in PBST, the detection of the relevant protein was assessed by enhanced chemiluminescence (Lite Ablot^®^ TURBO; Euro Clone, Milan, Italy). Densitometric analysis of the bands, relative to CDK4, was performed using Image J Software v1.51 (NIH, Bethesda, MD, USA).

### 4.4. Real-Time PCR Analysis

GSCs under normoxic and hypoxic conditions at different time points were collected and washed. TRI Reagent^®^ was added to cells and lysed using a syringe. Lysates were then centrifuged (12,000 rpm/10 min/4 °C). One fifth of the initial TRI Reagent^®^ volume of chloroform, has been added for 5 min at room temperature. After another centrifugation, three different section have been obtained, and RNA in the supernatant has been separated by the other components and transferred in another 1.5 mL tube. Subsequently, glycogen is added to the supernatant together with half of the initial TRI Reagent^®^ volume of isopropyl alcohol (isopropanol) at room temperature for 10 min. After centrifugation, the pellet has been washed with ethanol to obtain pure RNA. Following ethanol evaporation, RNA pellet has been resuspended in bidistilled water (ddH2O) and quantified by NanoDrop 2000 Spectrophotometer (Thermo Fisher Scientific, Rome, Italy). Preamplification system was used to reverse transcribe total RNA (1 µg) into complementary DNA according to manufacturer’s instructions (Invitrogen, Milan, Italy). Real time (RT-PCR) amplification was carried out in the 7000 Real-time PCR system (Applied Biosystems, Foster City, CA, USA) using Real time SyBr Green qPCR Superscript (Invitrogen, Milan, Italy). All reactions were carried out in triplicate.

### 4.5. Immunofluorescence Assays

GSCs, treated as described above, were collected in 15 mL tubes using a plastic monouse Pasteur pipette and washed in cold PBS. GSCs were washed and centrifuged three times at 4 °C to remove all the medium. Paraformaldehyde 3.7% was then added for 24 h at 4 °C. Subsequently, paraformaldehyde was removed and cells were washed twice with cold PBS. Permeabilization was obtained using 1% Triton^®^ X-100 (Sigma Aldrich) for 10 min and after two additional washes, cells were incubated with RAGE (Santa Cruz Biotechnology) primary antibody for 24 h at 4 °C. Subsequently, cells were washed for three times and incubated with secondary antibody Alexa Fluor^®^ 488 or Alexa Fluor^®^ 555 for 2 h. After two additional washes in PBS, cells were resuspended in 70% glycerol and mounted on frosted microscope slides, ground 90° (Thermo Scientific). Fluorescence was observed by LSM 510 confocal microscope (Zeiss, Varese, Italy).

### 4.6. Cellular Invasion

Invasion of GSCs was determined using the Cell Invasion Assay (Chemicon, Milan, Italy) and following manufacturer’s instructions. Briefly, the assay is composed of an Invasion Chamber, a 24-well tissue culture plate, with 12 cell culture inserts. The insert contains 8 µm pore size polycarbonate membrane, over which a thin layer of ECMatrix™ is dried. The ECM layer occludes the membranes pores, blocking non-invasive cells from migrating through. Invasive cells on the other hand, migrate through the ECM layer and cling to the bottom of polycarbonate membrane. Initially, 300 µL of warm serum free medium have been added to rehydrate inserts for about 2 h. After rehydration, medium has been gently removed from each insert and substituted with serum free medium containing 150,000 cells/point with or without 100 nM digoxin or 5 µM acriflavine for GSC #1, and 200 nM digoxin or 10 µM acriflavine for GSC #83. At the same time, serum free medium with or without necrotic extracts 1 µg/mL has been added to the bottom of the well. Hypoxic plates have been quickly put inside the hypoxic chamber for 24 h. Subsequently, medium has been removed and the ECM has been cleaned by using cotton swab. Invading cells have been stained by placing the insert into 500 µL of a staining solution for 20 min. Subsequently, inserts have been dipped in a beaker of water several times to remove the dye in excess. Cellular invasiveness has been quantified by dissolving stained cells in 10% acetic acid and transferring a consistent amount of the dye/solute mixture to a 96-well plate reader (Promega, Milan, Italy) for colorimetric reading of OD at 560 nm.

### 4.7. Co-Culture Experiments

For these experiments we used GFP-expressing GSCs. Initially, HUVECs have been collected and resuspended in an appropriate volume and stained with Calcein Red-Orange AM (Invitrogen). Cells were plated on coverslips in growth medium for 24 h in the presence or absence of necrotic extracts (1 µg/mL). Meanwhile, GFP-GSCs have been treated with digoxin, or acriflavine in normoxic and hypoxic conditions for 24 h. After this time, GFP-GSCs have been collected and plated in the same wells with HUVEC for 24 h. Then, cells have been collected and washed. Glasses have been washed twice and then mounted on microscope slides. Fluorescence was observed by LSM 510 confocal microscope (Zeiss).

### 4.8. Measurement of Intracellular ROS

To determine the amount of ROS production, 2′,7′-dichlorfluorescein-diacetate (DCFH-DA) was used. Following treatment cells were harvested by centrifugation at 1200 rpm for 5 min at room temperature. The pellets were resuspended in 0.5 mL of cell culture media containing 50 µM DCFH-DA and incubated for 30 min at 37 °C in 5% CO_2_. Fluorescence intensity was measured by flow cytometry (FACScalibur, BD Biosciences, San Jose, CA, USA).

### 4.9. In Vivo Experiments

Animal experiments have been performed in accordance to relevant institutional and national regulations. Initially, we tested the efficacy of necrotic extracts to promote the inflammation by injecting four different concentrations of necrotic extracts (5 ng/5 µL, 30 ng/5 µL, 60 ng/5 µL, 100 ng/5 µL) in the brain of NOD/SCID mice (n, 6; 4–6 weeks of age; CD1 NOD-/SCID mice, Charles Rives, Italy). Two mice, used as control, underwent a simple injection of the saline hypotonic buffer. Before grafting, mice were anesthetized with intraperitoneal injection of diazepam (2 mg/100 g) followed by intramuscular injection of ketamine (4 mg/100 g). The animal skulls were immobilized in a stereotactic head frame and a burr hole will be made 2 mm right of the midline and 1 mm anterior to the coronal suture, and cells slowly injected with a 10-µL Hamilton microsyringe placed at a depth of 3.5 mm from the dura. The animal which received the highest dose of necrotic extracts (100 ng/5 µL) did not tolerate treatment and were euthanized. After 2 weeks, the remaining mice were deeply anesthetized and transcardially perfused with 0.1 M PBS (pH = 7.4), followed by 4% paraformaldehyde in 0.1 M PBS. After perfusion, the brains were removed from the skull and post-fixed in the same fixative overnight at 4 °C. After rinsing in phosphate buffer, brains were cryoprotected in 15% sucrose solution followed by a passage in 30% sucrose solution both overnight at 4 °C. Free-floating sections were then analyzed for immunofluorescence. Sections were incubated in 10% normal horse serum in phosphate buffer containing 0.2% Triton X-100 for 30 min to reduce non-specific binding. These sections were then incubated with primary antibodies (GFAP, Chemicon, Milan, Italy; Iba1 Biocare Medical, Milan, Italy; β-Tubulin III Merck/Millipore, Milan, Italy) diluted in phosphate buffer containing 2% normal horse serum and 0.2% Triton X-100, overnight at 4 °C. After rinsing, the sections were incubated with labeled secondary antibodies (Alexa Fluor Molecular Probes, Eugene, OR, USA) for 1 h at RT. After a thorough rinse, the sections were incubated in phosphate buffer containing a Hoechst for 10 min at RT; sections were mounted on slides and coverslipped with antifade medium. Images were obtained with a Laser Scanning Confocal Microscope (Olympus FluoView FV1000, Olympus Inc, Melville, NY, USA). Images were treated, analyzed and brain slices reconstructed by using Adobe Photoshop software and fluorescence quantitation of histogram for the green channel was performed with ImageJ. To assess whether necrotic extracts may stimulate migration of GSCs in established brain xenografts, mice were first inoculated with GFP-GSCs following the procedure described above, 8 weeks later we inoculated necrotic extracts in the contralateral brain hemisphere. Control mice received intracerebral injection of hypotonic solution. Two weeks later, mice were sacrificed, their brains collected and analyzed as described above.

### 4.10. Statistical Analysis

Statistically significant differences have been tested with the Student’s paired *t* test. *p*-values less than 0.05 have been considered significant. All experiments have been performed in triplicate.

## 5. Conclusions

In this research paper, using in vitro and in vivo systems, we report that hypoxia and necrosis, present in the tumor microenvironment, represent two fundamental stimuli for glioblastoma stem cells (GSCs) migration. In fact, we showed that, in vitro, GSCs respond to hypoxia by increasing the expression of HIF-1α and of alarmin receptor RAGE. Such a response, in turn, increases GSC migration in the presence of necrotic stimulus. Moreover, such a situation is replicated in vivo where GSCs can migrate from a brain hemisphere to the other in which a necrotic extract has been injected. Finally, inhibition of the hypoxic response by digoxin and acriflavine prevented GSC response to hypoxia and migration. Our findings suggest that the modulation of both hypoxic response and inflammation may represent a valid strategy for reducing glioblastoma progression.

## Figures and Tables

**Table 1:** Mean fluorescent intensity per cell calculated by dividing total fluorescence intensity by the number of cells from at least three separate experiments including the one shown in Figure 4. *, significantly different from untreated sample. Significance was set at *p* < 0.05. C, control cells.

GSC #1		GSC #83	
Normoxia (mean fluorescence intensity per cell)		Normoxia (mean fluorescence intensity per cell)	
C	75.3 ± 6.5	C	50.7 ± 5.0
Digoxin 100 nM	70.7 ± 5.7	Digoxin 200 nM	56.7 ± 4.0
Acriflavine 5 µM	66.3 ± 5.7	Acriflavine 10 µM	60.3 ± 2.3
Hypoxia (mean fluorescence intensity per cell)		Hypoxia (mean fluorescence intensity per cell)	
C	92.3 ± 6.5	C	107 ± 9.8
Digoxin 100 nM	56.0 ± 4.6 *	Digoxin 200 nM	64.3 ± 4.7 *
Acriflavine 5 µM	32.0 ± 2.6 *	Acriflavine 10 µM	74.7 ± 4.6 *

## Supplementary Materials

Supplementary materials can be found at https://www.mdpi.com/1422-0067/21/8/2660/s1. Figure S1: Evaluation of Digoxin and Acriflavine dosage.

## Abbreviations

CSC	Cancer stem cell
GCS	glioblastoma stem-like cell
HIF	hypoxia inducible factor
PDH	prolyl hydroxylase
VHL	von Hippel-Lindau
ROS	Reactive Oxygen Species
HRE	Hypoxia Response Element
DAMP	damage associated molecular pattern
HMGB1	High Mobility Group Box 1
RAGE	Receptor for advanced glycation end products
TLR	Toll-like receptor
NF-kB	nuclear factor kappa-light-chain-enhancer of activated B cells
VEGF	vascular endothelial growth factor
HK2	Hexokinase 2
GFAP	Glial fibrillary acidic protein
IBA1	Ionized calcium binding adaptor molecule 1
GFP	green fluorescent protein
IRR	inflammatory reparative response

## References

[1] Hanahan D., Weinberg R.A. (2011). Hallmarks of cancer: The next generation. Cell.

[2] Hinshaw D.C., Shevde L.A. (2019). The Tumor Microenvironment Innately Modulates Cancer Progression. Cancer Res..

[3] Braun R.D., Lanzen J.L., Snyder S.A., Dewhirst M.W. (2001). Comparison of tumor and normal tissue oxygen tension measurements using OxyLite or microelectrodes in rodents. Am. J. Physiol. Heart Circ. Physiol..

[4] Hayashi Y., Yokota A., Harada H., Huang G. (2019). Hypoxia/pseudohypoxia-mediated activation of hypoxia-inducible factor-1α in cancer. Cancer Sci..

[5] Schito L., Semenza G.L. (2016). Hypoxia-Inducible Factors: Master Regulators of Cancer Progression. Trends Cancer..

[6] Kim J.W., Gao P., Liu Y.C., Semenza G.L., Dang C.V. (2007). Hypoxia-inducible factor 1 and dysregulated c-Myc cooperatively induce vascular endothelial growth factor and metabolic switches hexokinase 2 and pyruvate dehydrogenase kinase 1. Mol. Cell Biol..

[7] Mathupala S.P., Ko Y.H., Pedersen P.L. (2006). Hexokinase II: Cancer’s double-edged sword acting as both facilitator and gatekeeper of malignancy when bound to mitochondria. Oncogene.

[8] Vartanian A., Agnihotri S., Wilson M.R., Burrell K.E., Tonge P.D., Alamsahebpour A., Jalali S., Taccone M.S., Mansouri S., Golbourn B. (2016). Targeting hexokinase 2 enhances response to radio-chemotherapy in glioblastoma. Oncotarget..

[9] Baek J.H., Mahon P.C., Oh J., Kelly B., Krishnamachary B., Pearson M., Chan D.A., Giaccia A.J., Semenza G.L. (2005). OS-9 interacts with hypoxia-inducible factor 1alpha and prolyl hydroxylases to promote oxygen-dependent degradation of HIF-1alpha. Mol Cell..

[10] Semenza G.L. (2007). Hypoxia-inducible factor 1 (HIF-1) pathway. Sci STKE.

[11] Semenza G.L. (2010). Oxygen homeostasis. Wiley Interdiscip. Rev. Syst. Biol. Med..

[12] Huang J., Xie Y., Sun X., Zeh H.J., Kang R., Lotze M.T., Tang D. (2015). DAMPs, ageing, and cancer: The ‘DAMP Hypothesis’. Ageing Res. Rev..

[13] Hernandez C., Huebener P., Schwabe R.F. (2016). Damage-associated molecular patterns in cancer: A double-edged sword. Oncogene.

[14] Patel S. (2018). Danger-Associated Molecular Patterns (DAMPs): The Derivatives and Triggers of Inflammation. Curr. Allergy Asthma Rep..

[15] Kwak T., Drews-Elger K., Ergonul A., Miller P.C., Braley A., Hwang G.H., Zhao D., Besser A., Yamamoto Y., Yamamoto H. (2017). Targeting of RAGE-ligand signaling impairs breast cancer cell invasion and metastasis. Oncogene.

[16] Leclerc E., Vetter S.W. (2015). The role of S100 proteins and their receptor RAGE in pancreatic cancer. Biochim. Biophys. Acta.

[17] Tóbon-Velasco J.C., Cuevas E., Torres-Ramos M.A. (2014). Receptor for AGEs (RAGE) as mediator of NF-kB pathway activation in neuroinflammation and oxidative stress. CNS Neurol. Disord. Drug Targets.

[18] Tafani M., Di Vito M., Frati A., Pellegrini L., De Santis E., Sette G., Eramo A., Sale P., Mari E., Santoro A. (2011). Pro-inflammatory gene expression in solid glioblastoma microenvironment and in hypoxic stem cells from human glioblastoma. J. Neuroinflammat..

[19] Russo M.A., Sansone L., Carnevale I., Limana F., Runci A., Polletta L., Perrone G.A., De Santis E., Tafani M. (2015). One Special Question to Start with: Can HIF/NFkB be a Target in Inflammation?. Endocr. Metab. Immu. Disord. Drug Targets.

[20] Rider P., Kaplanov I., Romzova M., Bernardis L., Braiman A., Voronov E., Apte R.N. (2012). The transcription of the alarmin cytokine interleukin-1 alpha is controlled by hypoxia inducible factors 1 and 2 alpha in hypoxic cells. Front. Immunol..

[21] Muz B., de la Puente P., Azab F., Azab A.K. (2015). The role of hypoxia in cancer progression, angiogenesis, metastasis, and resistance to therapy. Hypoxia.

[22] Nassar D., Blanpain C. (2016). Cancer Stem Cells: Basic Concepts and Therapeutic Implications. Annu. Rev. Pathol..

[23] Carnero A., Lleonart M. (2016). The hypoxic microenvironment: A determinant of cancer stem cell evolution. Bioessays.

[24] Nandy S.B., Lakshmanaswamy R. (2017). Cancer Stem Cells and Metastasis. Prog. Mol. Biol. Transl. Sci..

[25] Singh S.K., Clarke I.D., Terasaki M., Bonn V.E., Hawkins C., Squire J., Dirks P.B. (2003). Identification of a cancer stem cell in human brain tumors. Cancer Res..

[26] O’Flaherty J.D., Gray S., Richard D., Fennell D., O’Leary J.J., Blackhall F.H., O’Byrne K.J. (2012). Circulating tumour cells, their role in metastasis and their clinical utility in lung cancer. Lung Cancer.

[27] Dhawan P., Ahmad R., Srivastava A.S., Singh A.B. (2011). Cancer stem cells and colorectal cancer: An overview. Curr. Top Med. Chem..

[28] Ahmed N., Abubaker K., Findlay J.K. (2014). Ovarian cancer stem cells: Molecular concepts and relevance as therapeutic targets. Mol. Aspects Med..

[29] Hamada S., Masamune A., Takikawa T., Suzuki N., Kikuta K., Hirota M., Hamada H., Kobune M., Satoh K., Shimosegawa T. (2012). Pancreatic stellate cells enhance stemcell-like phenotypes in pancreatic cancer cells. Biochem. Biophys. Res. Commun..

[30] Lang S.H., Frame F.M., Collins A.T. (2009). Prostate cancer stem cells. J. Pathol..

[31] Malaguarnera R., Morcavallo A., Giuliano S., Belfiore A. (2012). Thyroid cancer development and progression: Emerging role of cancer stem cells. Minerva Endocrinol..

[32] Lorico A., Bratbak D., Meyer J., Kunke D., Krauss S., Plott W.E., Solodushko V., Baum C., Fodstad O., Rappa G. (2005). Gamma-glutamylcysteine synthetase and L-buthionine-(S,R)-sulfoximine: A new selection strategy for gene-transduced neural and hematopoietic stem/progenitor cells. Hum. Gene Ther..

[33] Dunn G.P., Rinne M.L., Wykosky J., Genovese G., Quayle S.N., Dunn I.F., Agarwalla P.K., Chheda M.G., Campos B., Wang A. (2012). Emerging insights into the molecular and cellular basis of glioblastoma. Genes Dev..

[34] Singh S.K., Hawkins C., Clarke I.D., Squire J.A., Bayani J., Hide T., Henkelman R.M., Cusimano M.D., Dirks P.B. (2004). Identification of human brain tumour initiating cells. Nature.

[35] Pallini R., Ricci-Vitiani L., Banna G.L., Signore M., Lombardi D., Todaro M., Stassi G., Martini M., Maira G., Larocca L.M. (2008). Cancer stem cell analysis and clinical outcome in patients with glioblastoma multiforme. Clin. Cancer Res..

[36] Marziali G., Signore M., Buccarelli M., Grande S., Palma A., Biffoni M., Rosi A., D’Alessandris Q.G., Martini M., Larocca L.M. (2016). Metabolic/Proteomic Signature Defines Two Glioblastoma Subtypes With Different Clinical Outcome. Sci. Rep..

[37] Günther H.S., Schmidt N.O., Phillips H.S., Kemming D., Kharbanda S., Soriano R., Modrusan Z., Meissner H., Westphal M., Lamszus K. (2008). Glioblastoma-derived stem cell-enriched cultures form distinct subgroups according to molecular and phenotypic criteria. Oncogene.

[38] D’Alessandris Q.G., Biffoni M., Martini M., Runci D., Buccarelli M., Cenci T., Signore M., Stancato L., Olivi A., De Maria R. (2017). The clinical value of patient-derived glioblastoma tumorspheres in predicting treatment response. Neuro Oncol..

[39] Papale M., Ferretti E., Battaglia G., Bellavia D., Mai A., Tafani M. (2018). EZH2, HIF-1, and Their Inhibitors: An Overview on Pediatric Cancers. Front. Pediatr..

[40] Zhang H., Qian D.Z., Tan Y.S., Lee K., Gao P., Ren Y.R., Rey S., Hammers H., Chang D., Pili R. (2008). Digoxin and other cardiac glycosides inhibit HIF-1alpha synthesis and block tumor growth. Proc. Natl. Acad. Sci. USA.

[41] Lee K., Zhang H., Qian D.Z., Rey S., Liu J.O., Semenza G.L. (2009). Acriflavine inhibits HIF-1 dimerization, tumor growth, and vascularization. Proc. Natl. Acad. Sci. USA.

[42] Nobre A.R., Entenberg D., Wang Y., Condeelis J., Aguirre-Ghiso J.A. (2018). The Different Routes to Metastasis via Hypoxia-Regulated Programs. Trends Cell Biol..

[43] Tafani M., Sansone L., Limana F., Arcangeli T., De Santis E., Polese M., Fini M., Russo M.A. (2016). The Interplay of Reactive Oxygen Species, Hypoxia, Inflammation, and Sirtuins in Cancer Initiation and Progression. Oxid. Med. Cell Longev..

[44] Huang W.J., Chen W.W., Zhang X. (2016). Glioblastoma multiforme: Effect of hypoxia and hypoxia inducible factors on therapeutic approaches. Oncol Lett..

[45] Joseph J.V., Conroy S., Pavlov K., Sontakke P., Tomar T., Eggens-Meijer E., Balasubramaniyan V., Wagemakers M., den Dunnen W.F., Kruyt F.A. (2015). Hypoxia enhances migration and invasion in glioblastoma by promoting a mesenchymal shift mediated by the HIF1α-ZEB1 axis. Cancer Lett..

[46] Friedmann-Morvinski D. (2014). Glioblastoma heterogeneity and cancer cell plasticity. Crit. Rev. Oncog..

[47] Seyfried T.N., Kiebish M.A., Marsh J., Shelton L.M., Huysentruyt L.C., Mukherjee P. (2011). Metabolic management of brain cancer. Biochim. Biophys. Acta.

[48] Semenza G.L. (2013). HIF-1 mediates metabolic responses to intratumoral hypoxia and oncogenic mutations. J. Clin. Investig..

[49] Chen F., Chen L., He H., Huang W., Zhang R., Li P., Meng Y., Jiang X. (2016). Up-regulation of microRNA-16 in Glioblastoma Inhibits the Function of Endothelial Cells and Tumor Angiogenesis by Targeting Bmi-1. Anticancer Agents Med. Chem..

[50] Xiao B., Zhou X., Ye M., Lv S., Wu M., Liao C., Han L., Kang C., Zhu X. (2016). MicroRNA-566 modulates vascular endothelial growth factor by targeting Von Hippel-Landau in human glioblastoma in vitro and in vivo. Mol. Med. Rep..

[51] Grande S., Palma A., Ricci-Vitiani L., Luciani A.M., Buccarelli M., Biffoni M., Molinari A., Calcabrini A., D’Amore E., Guidoni L. (2018). Metabolic Heterogeneity Evidenced by MRS among Patient-Derived Glioblastoma Multiforme Stem-Like Cells Accounts for Cell Clustering and Different Responses to Drugs. Stem Cells Int..

[52] Oudin M.J., Weaver V.M. (2016). Physical and Chemical Gradients in the Tumor Microenvironment Regulate Tumor Cell Invasion, Migration, and Metastasis. Cold Spring Harb. Symp. Quant. Biol..

[53] Taddei M.L., Giannoni E., Comito G., Chiarugi P. (2013). Microenvironment and tumor cell plasticity: An easy way out. Cancer Lett..

[54] Palanissami G., Paul S.F.D. (2018). RAGE and Its Ligands: Molecular Interplay Between Glycation, Inflammation, and Hallmarks of Cancer-a Review. Horm. Cancer.

[55] Tafani M., Schito L., Pellegrini L., Villanova L., Marfe G., Anwar T., Rosa R., Indelicato M., Fini M., Pucci B. (2011). Hypoxia-increased RAGE and P2X7R expression regulates tumor cell invasion through phosphorylation of Erk1/2 and Akt and nuclear translocation of NF-{kappa}B. Carcinogenesis.

[56] Gopal P., Gosker H.R., Theije C.C., Eurlings I.M., Sell D.R., Monnier V.M., Reynaert N.L. (2015). Effect of chronic hypoxia on RAGE and its soluble forms in lungs and plasma of mice. Biochim. Biophys. Acta.

[57] Tafani M., Russo A., Di Vito M., Sale P., Pellegrini L., Schito L., Gentileschi S., Bracaglia R., Marandino F., Garaci E. (2010). Up-regulation of pro-inflammatory genes as adaptation to hypoxia in MCF-7 cells and in human mammary invasive carcinoma microenvironment. Cancer Sci..

[58] Pichiule P., Chavez J.C., Schmidt A.M., Vannucci S.J. (2007). Hypoxia-inducible factor-1 mediates neuronal expression of the receptor for advanced glycation end products following hypoxia/ischemia. J. Biol. Chem..

[59] Franses J.W., Drosu N.C., Gibson W.J., Chitalia V.C., Edelman E.R. (2013). Dysfunctional endothelial cells directly stimulate cancer inflammation and metastasis. Int. J. Cancer.

[60] Mamlouk S., Wielockx B. (2013). Hypoxia-inducible factors as key regulators of tumor inflammation. Int. J. Cancer.

[61] Grivennikov S.I., Karin M. (2010). Inflammation and oncogenesis: A vicious connection. Curr. Opin. Genet. Dev..

[62] Wang G., Li Y., Yang Z., Xu W., Yang Y., Tan X. (2018). ROS mediated EGFR/MEK/ERK/HIF-1α Loop Regulates Glucose metabolism in pancreatic cancer. Biochem. Biophys. Res. Commun..

[63] Movafagh S., Crook S., Vo K. (2015). Regulation of hypoxia-inducible factor-1a by reactive oxygen species: New developments in an old debate. J. Cell Biochem..

[64] Guidoni L., Ricci-Vitiani L., Rosi A., Palma A., Grande S., Luciani A.M., Pelacchi F., di Martino S., Colosimo C., Biffoni M. (2014). 1H NMR detects different metabolic profiles in glioblastoma stem-like cells. NMR Biomed..

[65] Yang Z., Wang K.K. (2015). Glial fibrillary acidic protein: From intermediate filament assembly and gliosis to neurobiomarker. Trends Neurosci..

[66] Hoogland I.C., Houbolt C., van Westerloo D.J., van Gool W.A., van de Beek D. (2015). Systemic inflammation and microglial activation: Systematic review of animal experiments. J. Neuroinflammat..

[67] Lin J.C., Tsai J.T., Chao T.Y., Ma H.I., Liu W.H. (2019). Musashi-1 Enhances Glioblastoma Migration by Promoting ICAM1 Translation. Neoplasia.

[68] Alieva M., Margarido A.S., Wieles T., Abels E.R., Colak B., Boquetale C., Jan Noordmans H., Snijders T.J., Broekman M.L., van Rheenen J. (2017). Preventing inflammation inhibits biopsy-mediated changes in tumor cell behavior. Sci. Rep..

[69] Alieva M., van Rheenen J., Broekman M.L.D. (2018). Potential impact of invasive surgical procedures on primary tumor growth and metastasis. Clin. Exp. Metastasis.

