# Supplementary material for: Hypoxia, Inflammation and Necrosis as Determinants of Glioblastoma Cancer Stem Cells Progression
Source: Int J Mol Sci. 2020 Apr 11;21(8):2660. doi: 10.3390/ijms21082660 (PMC7215563; doi:10.3390/ijms21082660)
Supplement: Supplementary file 1 [file ijms-21-02660-s001.pdf]

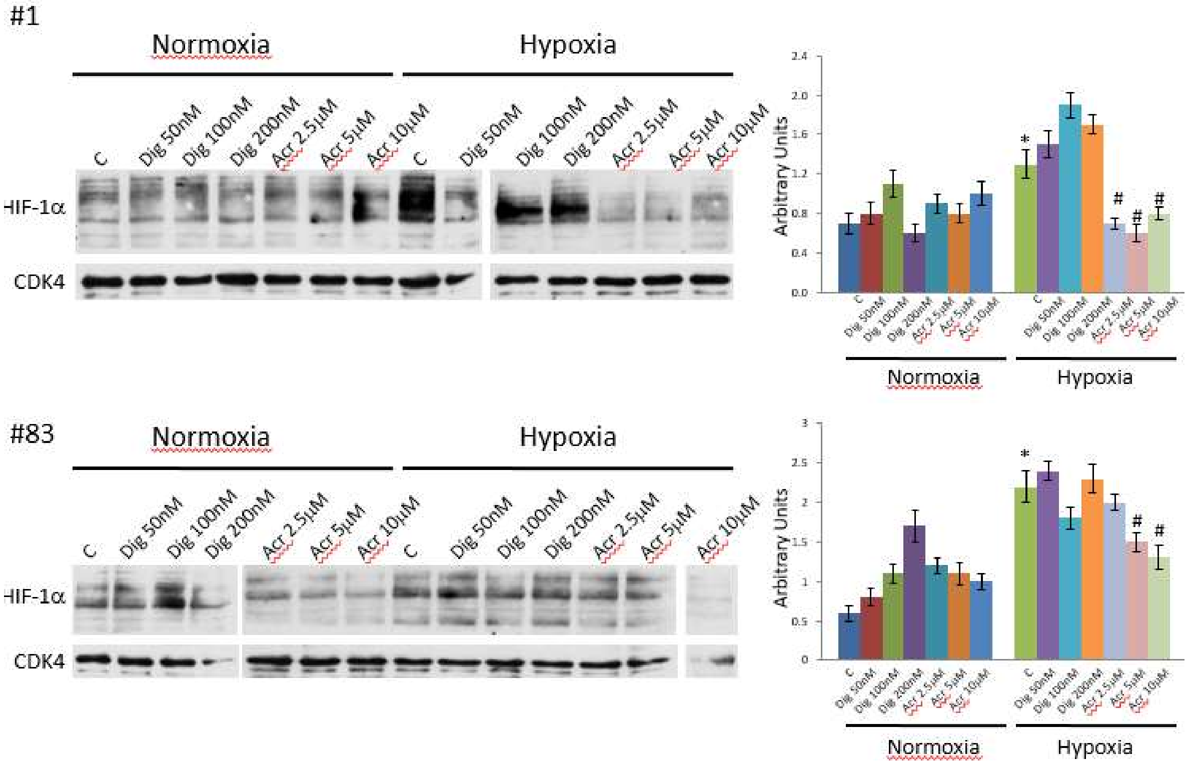

Figure S1. Evaluation of Digoxin and Acriflavine dosage. GSC#1 and 83 were kept under normoxic or hypoxic condition in the presence or absence of different doses of Digoxin (Dig) or Acriflavine (Acr). for 24h. HIF-1a expression was evaluated by Western blot assay as described in Materials and Methods. CDK4 was used as loading control. Densitometric analysis of the gels was performed as described in Materials and Methods. Data are representative of three separate experiments.* Significantly different from untreated normoxic cells. #Significantly different from untreated hypoxic cells. Significance was set at $p < 0.05$.
